# Diagnostic value of the combined test of leukocytes in urine and TB-DOT and T-SPOT.TB in blood for urinary tuberculosis

**DOI:** 10.3389/fmicb.2025.1535490

**Published:** 2025-03-05

**Authors:** Yanyan Li, Yachun Wang, Lukuan Wei, Wei Wang

**Affiliations:** ^1^Medical Laboratory, Henan Provincial Chest Hospital of Zhengzhou University, Zhengzhou, China; ^2^Henan Provincial Key Laboratory of Tuberculosis Diagnostic Medicine, Zhengzhou, China; ^3^Henan Provincial Infectious Diseases (Tuberculosis) Clinical Medical Research Center, Zhengzhou, China; ^4^Henan Provincial Science and Technology Department “International Joint Laboratory of Tuberculosis”, Zhengzhou, China; ^5^Henan Provincial Development and Reform Commission “Multidrug-Resistant Tuberculosis Detection and Treatment” Engineering Research Center, Zhengzhou, China

**Keywords:** urinary tuberculosis, non-urinary tuberculosis, white blood cell, tuberculosis antibody test, T-cell spot tests for tuberculosis infection, creatinine

## Abstract

**Background:**

This study aims to evaluate the clinical utility of routine urinary tests and renal function assessments, as well as the Tuberculosis antibody test (TB-DOT) and T-cell spot tests for TB infection (T-SPOT.TB), either individually or in combination, for diagnosing urinary tuberculosis (UTB).

**Methods:**

We conducted a retrospective analysis of urinary routine tests, renal function tests, TB-DOT, and T-SPOT.TB—administered alone or in combination—in 95 patients suspected of having UTB from January 2020 to December 2022 at our institution.

**Results:**

Significant differences were observed in the levels or positivity of white blood cells (WBC), red blood cells (RBC), creatinine (Crea), TB-DOT, and T-SPOT.TB between the UTB group and the non-UTB group (*P* < 0.05). Among the individual tests, T-SPOT.TB exhibited the highest specificity and positive predictive value (PPV), while WBC demonstrated the highest area under the curve (AUC). Both TB-DOT and RBC showed relatively good sensitivity. Additionally, WBC levels correlated with both TB-DOT and T-SPOT.TB results. The combination of WBC, TB-DOT, and T-SPOT.TB provided the best sensitivity, negative predictive value (NPV), and AUC when evaluated in parallel with the other tests.

**Conclusion:**

For the early identification of UTB, the sensitivity of T-SPOT.TB and TB-DOT tests is superior to that of routine urinary and renal function tests. The parallel combination of WBC, TB-DOT, and T-SPOT.TB offers enhanced diagnostic efficacy for UTB, facilitating rapid clinical diagnosis.

## Introduction

Urinary tuberculosis (UTB) is a chronic, progressive disease caused by *Mycobacterium tuberculosis* (MTB). It is primarily caused by an MTB infection in the urinary system, which can spread through the bloodstream from initial lung lesions (Rajpurohit et al., 2023). According to estimates, developing countries, particularly China, account for more than 90% of extrapulmonary tuberculosis (EPTB) cases (Abbara and Davidson, 2011). UTB is one of the most prevalent types of EPTB, accounting for 30%−40% of cases (Harding, 2020; Figueiredo et al., 2017). Unfortunately, treatment success rates for UTB are low. Timely and precise diagnosis and treatment are critical because they can halt the progression and spread of UTB lesions and assist avert potentially deadly consequences (Kroidl et al., 2015). Thus, early detection of UTB is critical for effective prevention and therapy.

UTB lesions first impact specific areas of the urinary system, causing few clinical symptoms and only atypical results on urine testing. Urine culture for MTB is the traditional “gold standard” for diagnosis (Simner et al., 2018), but it is less effective for early UTB detection. This is because solid cultures require long detection times, while liquid cultures require expensive equipment and have a relatively low sensitivity (Sallami et al., 2014; Drain et al., 2014). Furthermore, positivity for MTB diagnosis has low sensitivity and fails to distinguish between non-MTB mycobacteria such as *Mycobacterium leprae, Nocardia species*, and *Legionella species* (Jiang et al., 2016; Haldar et al., 2011). The Xpert MTB/RIF assay is useful, but it is too expensive to meet the demand for low-cost UTB treatment and clinical diagnosis (Chen et al., 2020; Weyer et al., 2013). Ultrasound, X-ray, and other imaging procedures have reduced diagnostic accuracy and precision (Ye et al., 2016). In developing countries, a quick and inexpensive diagnostic test could be useful. In this case, urine parameter assessment is a useful approach strategy.

Currently, basic, rapid, and inexpensive laboratory tests are frequently used in patients with suspected UTB, including regular urine and renal function tests, as well as T-cell spot assays for TB infection (T-SPOT.TB) and TB antibody tests (TB-DOT). Most people with UTB will have a high concentration of white blood cells (WBC) and red blood cells (RBC) in their urine, as well as acidic urine; however, TB cannot be detected merely by doing a regular urine test. UTB predominantly damages the renal parenchyma, causing kidney units to vanish and reduced function, resulting in elevated urea, creatinine (Crea), and uric acid (UA) levels. For individuals with UTB, routine urine tests and renal function tests are simply routine diagnostics, but peripheral blood T-SPOT.TB.

Therefore, from 2020 to 2022, laboratory results of renal function, TB-DOT, T-SPOT.TB, and urine routine were collected from UTB patients in our hospital with an emphasis on statistical analysis. The sensitivity of the T-SPOT.TB or TB-DOT test is significantly higher in the early detection of UTB than that of standard urine and renal function testing. WBC combined with T-SPOT and TB-DOT. In addition to being a quick and early adjunct test for clinical usage in UTB diagnosis, TB can greatly enhance UTB diagnosis.

## Methods

### Research subjects

**Figure 1:**
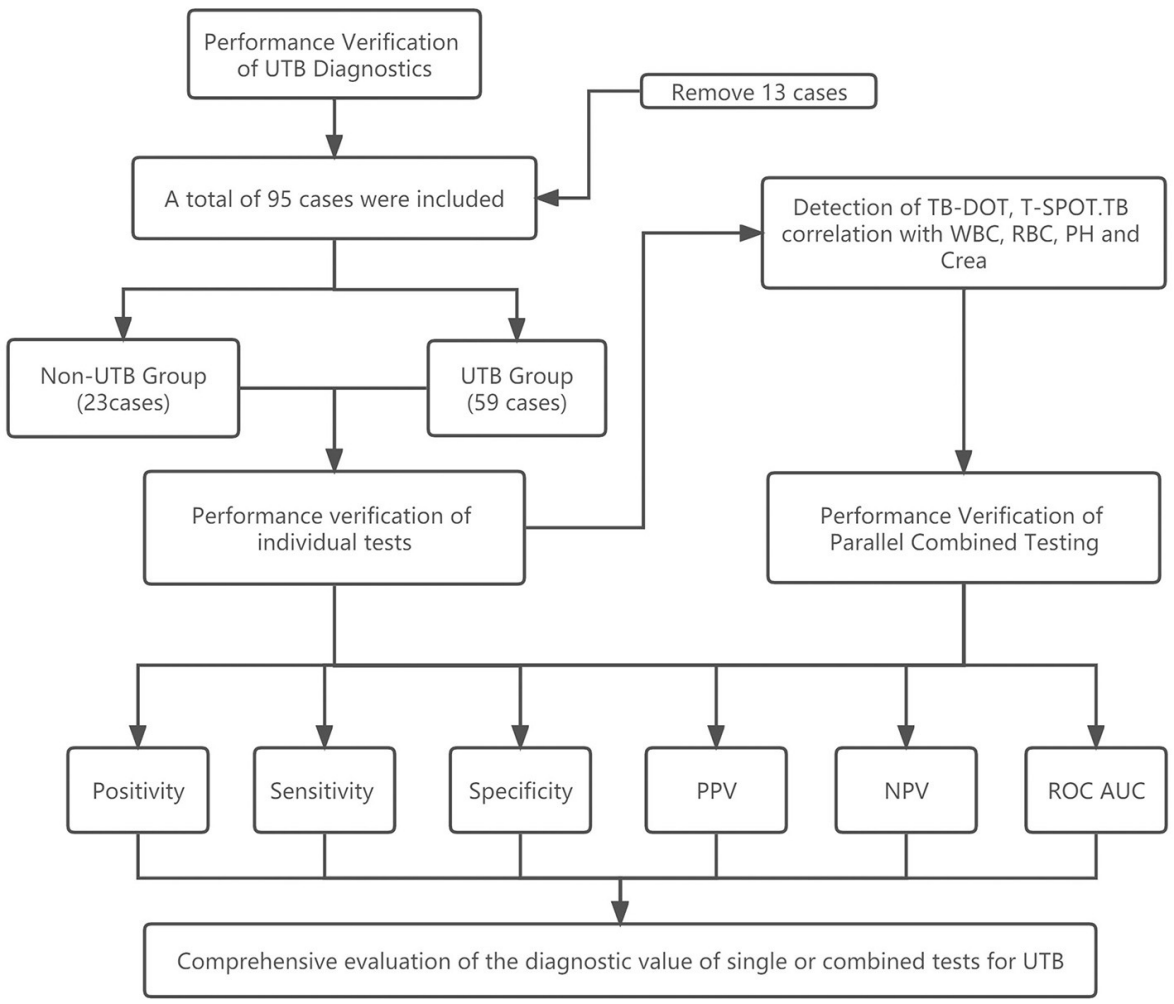
Flowchart of this study.

The study examined the urine routine, renal function, TB-DOT antibodies, and T-SPOT.TB testing of 95 suspected UTB patients who were examined in the laboratory medicine department of Henan Chest Hospital between January 2020 and December 2022. Thirteen of their patients since they didn't fit the criteria (see Figure 1).

### Inclusion and exclusion criteria

Inclusion criteria: (1) Patients with UTB treated in our hospital. (2) The hospital maintains the patient's complete clinical information. (3) Patients with pathologically proven or empirical treatment and clinical symptoms in remission. (4) The patient's informed consent form was signed.

Exclusion criteria: Any one of the following, (1) Patients who have antibodies to the human immunodeficiency virus (HIV) that were found to be present. Reason for exclusion: Due to their underdeveloped immune system, HIV patients, infants, and children are less sensitive to T-SPOT.TB. Comparing TB testing with the general TB population. (2) Patients with unknown diagnosis of UTB. (3) Patients who, at the time of consultation, were under the age of 18.

### Routine urine test

Complete the test in 1 h by collecting fresh urine in a clean, dry container (it is advised to take first-morning urine). Before the test, the automatic urine dry chemistry analyzer is routinely cleaned, adjusted, and calibrated, and the operation stages are carried out following the instruction manual (AVE, Changsha, China). Normal reference range: RBC 0–6/μL, WBC 0–12/μL, potential of hydrogen (PH) 5–6.

### Renal function test

In this study, serum levels of Urea, Crea, and UA were measured with a fully automated biochemical analyzer (BECKMAN COULTER AU680) and reagents (Gcell, Beijing, China). Urea levels of 1.7–8.3 mmol/L by turbidimetric assay, Crea levels of 40–97 μmol/L by picric acid assay were considered normal, and UA levels of 150–440 μmol/L by UV rate assay were all considered normal.

### *Mycobacterium tuberculosis* IgG antibody (TB-DOT) test

The patient's peripheral venous blood was drawn into a procoagulant tube, spun at 3,000 rpm for 15 min, and the supernatant was collected and stored at 2°C−8°C for testing. Place the TB-DOT kit at room temperature, add 100 μL of the test serum into the test wells, and position it horizontally. Within 15–30 min, check the results; beyond 30 min, the results will be invalid. Result determination: (1) Negative: The control line alone was displayed; (2) Positive: The control line and the test line were both visible; (3) Reagent invalidation: No display of the control line. The experiment was carried out precisely as directed per the kit's instructions (Beier, Beijing, China).

### *Mycobacterium tuberculosis*-specific T-lymphocyte assay (T-SPOT.TB)

The patient's peripheral venous blood was drawn into heparin anticoagulation tubes, transferred, and kept at room temperature while the entire procedure was meticulously followed to the letter of the kit's instructions (Oxford Immunotec T-Spot TB Package Insert, 2010). After centrifugation, the supernatant was combined with RPMI 1640 (1:1), Ficoll lymphocyte isolate (1:2), and T-SPOT.TB brings the cell density to 2.5 × 106 cells/mL was added. The enzyme conjugate was incubated at 4°C for 1 h. By mixing in a color developer for 5–7 min, the color was created. To determine the number of spots per well, use a readable counter. Result determination: If there is a response to Antigen A and/or Antigen B and the test is “reactive,” refer to the following criteria: Blank control wells with 0–5 spots and (number of spots in antigen A or antigen B holes)—(number of spots in blank control holes) ≥6; If the number of spots in the blank control holes is 6–10 and (the number of spots in the antigen A or antigen B holes) ≥2 × (the number of spots in the blank control holes). If the above criteria are not met and the positive control holes are normal, the test result is “non-reactive.” The test result is deemed “inconclusive” and necessitates a second examination when the results of the positive control well are “inconclusive,” and the results of both the antigen A and antigen B holes are “non-reactive.”

### Testing index

In this investigation, routine urine tests (WBC, RBC, PH), renal function (Crea, Urea, UA), and TB-DOT or T-SPOT.TB were compared for their ability to detect UTB, as well as their sensitivity, specificity, PPV, NPV, and AUC. In addition, in the parallel combination test, all four tests, WBC, Crea, TB-DOT and T-SPOT.TB, were considered positive only if they were positive.

### Statistical analysis

The parameters were represented as means ± standard deviations ($\bar{x\;}\pm$ s) and the data were analyzed using the statistical program SPSS 25.0. The counts were expressed as rates using the χ^2^ split method. The *t*-test for independent samples was used to compare the two groups. one-way ANOVA was used for comparison between multiple groups. Pearson correlation analysis was performed and scatter plots, box plots, receiver operating characteristic (ROC) curves were plotted using Prism software. Optimal thresholds were determined based on the Jorden index (sensitivity + specificity 1). In addition, the optimal diagnostic thresholds and the corresponding sensitivity and specificity were also calculated for each test or parallel combination of tests. *P* < 0.05 is considered a statistically significant difference.

## Results

### General information about research subjects

In this study, 82 participants were categorized into the UTB and non-UTB groups. In the UTB group, there were 59 patients, including 29 males (49.2%, *P* > 0.05) and 30 females (50.8%, *P* > 0.05), with an average age of (47.25 ± 13.89) years. In the non-UTB group, there were 23 patients, of whom 10 (43.5%, *P* > 0.05) were male and 13 (56.5%, *P* > 0.05) were female, with a mean age of (45.21 ± 16.90) years. With 44 (74.6%, *P* < 0.05) in the UTB group over 30 and under 60 years of age and 14 (60.9%, *P* < 0.05) people in the non-UTB group over 30 and under 60, the age distribution was primarily middle-aged (see Table 1).

**Table 1:** General information of the research subjects (*n*, %).

**Group/index**		**Total**	**Non-UTB**	**UTB**	χ^**2**^ **test**		**OR [95% CI]**
					χ^2^	***P***	
Sex	Male	39	10 (43.5)	29 (49.2)	0.214	0.644	0.796 (0.302–2.098)
	Female	43	13 (56.5)	30 (50.8)			
Age	< 30 years	8	4 (17.4)	4 (6.8)	0.322	0.570	1.338 (0.489–3.666)
	30–60 years	58	14 (60.9)^①②^	44 (74.6)^①②^			
	>60 years	16	5 (21.7)	11 (18.6)			

① Compared to Age < 30 years, *P* < 0.05; ② Compared to Age >30 years, *P* < 0.05; UTB, urinary tuberculosis; Non-UTB, Non urinary tuberculosis; OR, odds ratio; CI, confidence interval.

### Comparison of the levels and positive rates of each test factor between the two groups of patients

**Figure 2:**
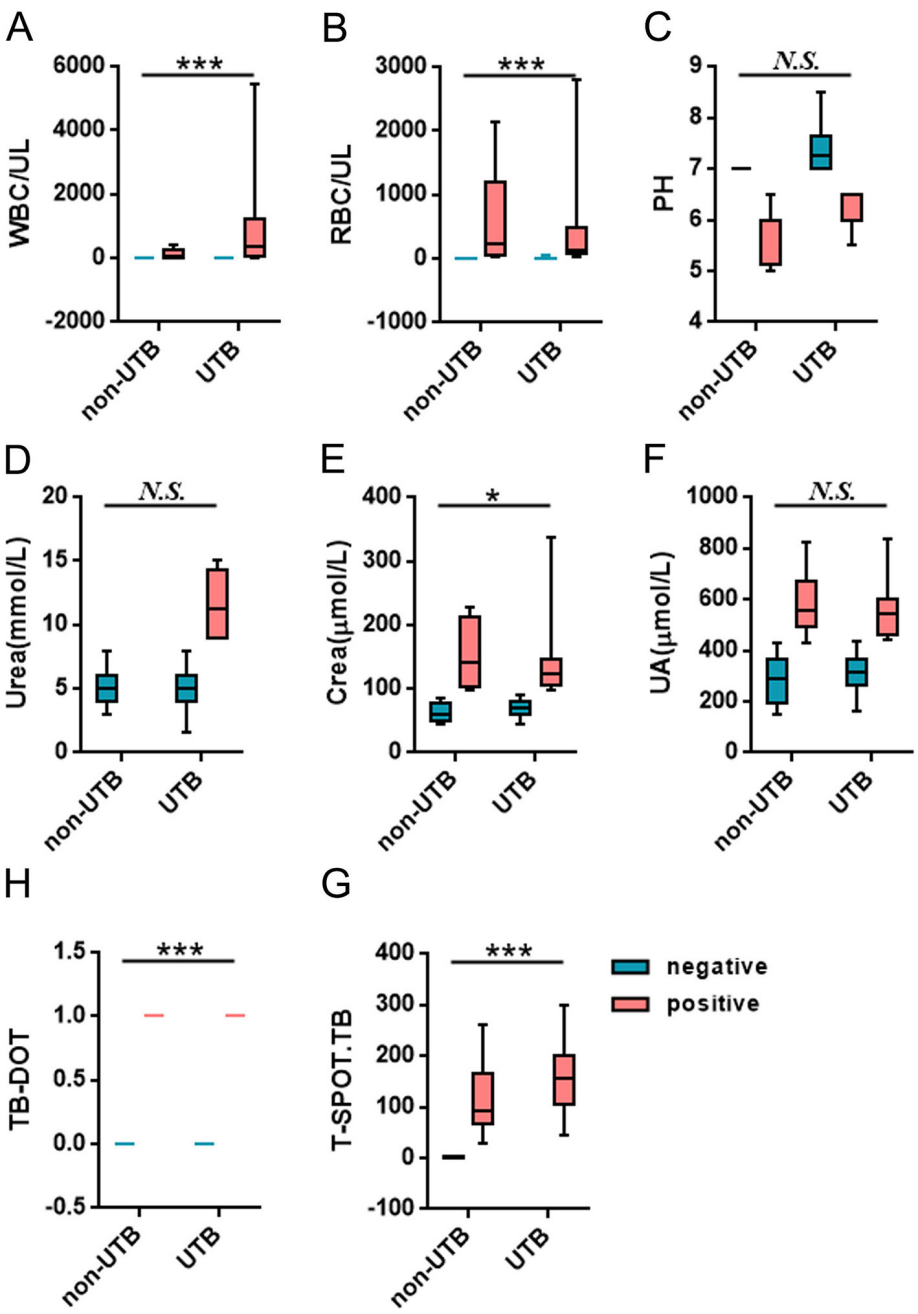
Comparison of the levels and positive rates of each test factor between the two groups of patients.

In the urine routine, the positive rates of WBC, RBC, and PH were 94.6%, 71.4%, and 82.1% in the UTB group. As compared to the non-UTB group, the positive rate of PH in the UTB group was not statistically significant and only slightly decreased (*P* < 0.05) (see Figure 2C and Table 2); but the positive rates of WBC and RBC in the UTB group were statistically considerably higher (*P* < 0.001), as shown in Figures 2A, B, and Table 2. In the renal function tests, the positive rates of Urea, Crea, and UA in the UTB group were 11.3%, 34.0%, and 34.0%, respectively, which were not statistically significant (*P* > 0.05) in comparison to the non-UTB group, as shown in Figures 2D, F, and Table 2; however, the positive rate of Crea was significantly higher (*P* < 0.05), as shown in Figure 2E and Table 2. Figures 2G, H, and Table 2 show the positive rates of T-SPOT.TB and TB-DOT in the UTB group were 87.88% and 100%, respectively. Both were significantly higher than those in the non-UTB group (*P* < 0.001).

**Table 2:** Comparison of the levels and positive rates of each test factor between the two groups of patients (*n*, %, $\bar{x}$ ± s).

**Examination/index**	**Total**	**Non-UTB**	**UTB**	χ^**2**^ **test**		**OR [95% CI]**
				χ^2^	***P***	
WBC/UL	79	12/23 (52.2)	53/56 (94.6)	20.353	0.001	196.1 [−1037 to −256.2]
		(71.0 ± 125.8)	56 (717.7 ± 933.5)^***^			
RBC/UL	79	5/23 (21.7)	40/56 (71.4)	0.836	0.261	113.2 [−353.6–97.12]
		23 (124.4 ± 447.2)	56 (252.6 ± 460.9)^***^			
PH	79	20/23 (87.0)	46/56 (82.1)	0.033	0.005	0.149 [−0.725 to −0.130]
		23 (5.96 ± 0.64)	56 (6.38 ± 0.59)			
Urea (umol/L)	76	0/23(–)	6/53 (11.3)	4.086	0.142	0.473 [−1.644–0.241]
		23 (4.96 ± 1.43)	53 (5.66 ± 2.67)			
Crea (mmol/L)	76	4/23 (17.4)	18/53 (34.0)	0.596	0.183	13.10 [−43.70–8.512]
		23 (78.43 ± 42.33)	53 (96.03 ± 56.22)^*^			
UA (mmol/L)	76	6/23 (26.1)	18/53 (34.0)	0.820	0.400	38.14 [−108.3–43.75]
		23 (357.04 ± 180.68)	53 (395.04 ± 143.70)			
TB-DOT	34	5 (5/16, 31.25%)	29 (29/33, 87.88)^***^	63.746	0.001	0.116 [−0.699 to −0.219]
T-SPOT.TB	69	14/21 (66.7)	48/48 (100.0)	0.022	< 0.001	20.29 [−124.8 to −43.77]
		21 (76.57 ± 78.26)	48 (160.83 ± 77.22)^***^			

^*^Compared to Non-UTB, ^*^P < 0.05, ^***^P < 0.001.

UTB, urinary tuberculosis; Non-UTB, non-urinary tuberculosis; T-SPOT.TB, T-cell spot tests for tuberculosis infection; TB-DOT, tuberculosis antibody test; Crea, creatinine; UA, uric acid; PH, potential of hydrogen; WBC, white blood cell; RBC, red blood cell; CI, confidence interval; OR, odds ratio.

### Comparison of the diagnostic efficacy of single tests for UTB

In routine urine testing, the sensitivity (88.9%) of RBC was higher, but its specificity (52.9%) and PPV (71.4%) were poor; the sensitivity (69.7%), specificity (23.1%), and NPV (13.0%) of PH were poor; although the sensitivity (81.5%) of WBC was lower than that of RBC, the specificity (78.6%) of WBC and PPV (94.6%) were better than those of RBC and PH. When performing renal function tests, we found that although the sensitivity (100%, 81.8%, and 75%) of Urea, Crea, and UA was high, their specificity (32.4%, 35.2%, and 32.7%) and PPV (11.3%, 34%, and 34%) were poor. Although the sensitivity of TB-DOT (85.7%) was higher than that of T-SPOT.TB (77.4%), the specificity and PPV of TB-DOT (78.6%, 90.9%) were lower than those of T-SPOT.TB (100%, 100%). The specificity and PPV of T-SPOT.TB was significantly higher and the NPV was significantly lower compared with Urea; the difference was statistically significant (*P* < 0.05); the specificity was significantly higher compared with PH, and the difference was statistically significant (*P* < 0.05) (see Table 3).

**Table 3:** Comparison of the diagnostic efficacy of single tests for UTB [*n*, %].

**Examination/index**	**Sensitivity**	**Specificity**	**PPV**	**NPV**	χ^**2**^ **test**		**OR [95% CI]**
					χ^2^	***P***	
WBC	81.5 (53/65)	78.6 (11/14)	94.6 (53/56)^②^	47.8 (11/23)	20.167	0.000	16.169 [3.906–67.138]
RBC	88.9 (40/45)	52.9 (18/34)	71.4 (40/56)	78.3 (18/23)	16.420	0.000	9.000 [2.856–28.366]
PH	69.7 (46/66)^②^	23.1 (3/13)	82.1 (46/56)	13.0 (3/23)	0.275	0.600	0.690 [0.171–2.778]
Urea	100.0 (6/6)	32.4 (23/71)	11.3 (6/53)	100.0 (23/23)^①^	2.827	0.093	0.671 [0.570–0.791]
Crea	81.8 (18/22)	35.2 (19/54)	34.0 (18/53)	82.6 (19/23)^①^	2.141	0.143	2.443 [0.722–8.265]
UA	75.0 (18/24)^②^	32.7 (17/52)	34.0 (18/53)	73.9 (17/23)	0.460	0.497	1.457 [0.490–4.336]
TB-DOT	85.7 (30/35)	78.6 (11/14)	90.9 (30/33)	68.8 (11/16)	18.793	0.000	22.000 [4.489–107.813]
T-SPOT.TB	77.4 (48/62)	100.0 (7/7)^①②^	100.0 (48/48)^②^	33.3 (7/21)^②^	40.145	0.000	0.127 [0.064–0.254]

^N.S^*P* > 0.05; ^*^P < 0.05, ^***^P < 0.001.

① Compared to PH, *P* < 0.05; ② Compared to Urea, *P* < 0.05.

UTB, urinary tuberculosis; Non-UTB, non-urinary tuberculosis; T-SPOT.TB, T-cell spot tests for tuberculosis infection; TB-DOT, tuberculosis antibody test; Crea, creatinine; UA, uric acid; PH, the potential of hydrogen; WBC, white blood cell; RBC, red blood cell; CI, confidence interval; OR, odds ratio; PPV, Positive predictive value; NPV, Negative predictive value.

**Figure 3:**
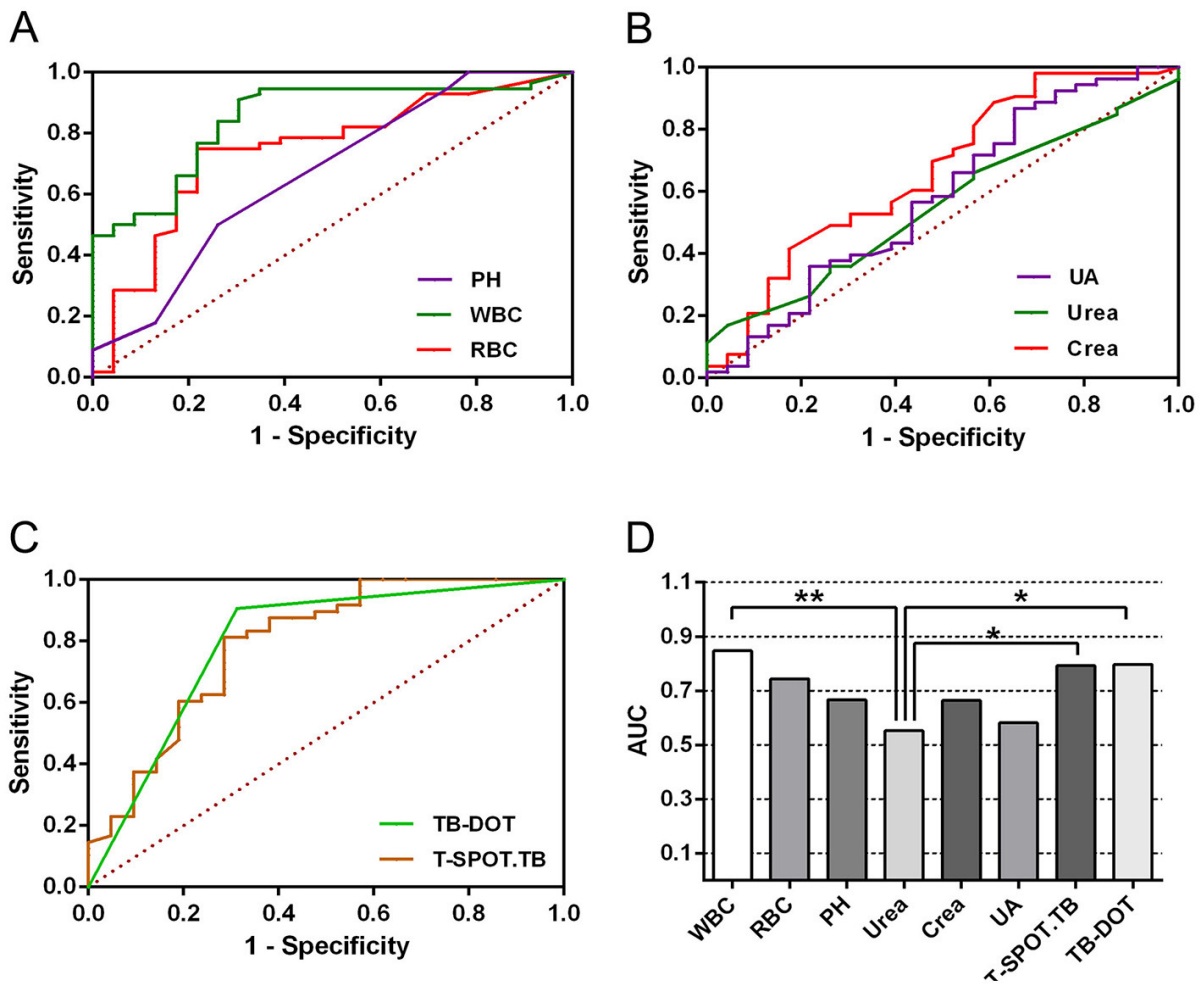
ROC curve for the diagnosis of UTB with a single test.

To further validate the diagnostic efficacy of single tests for UTB, ROC curve analysis was performed for each test in this subject, as shown in Table 4 and Figure 3. In urine routine, the AUCs of WBC, RBC, and PH were 0.847 (*P* < 0.001), 0.744 (*P* < 0.001) and 0.667 (*P* < 0.05), respectively; the sensitivities were 91.1%, 75.0%, and 50.0%, and the specificities were 69.6%, 78.3%, and 73.9%, respectively, all of which were statistically significant, as shown in Figure 3A and Table 4. In the renal function test, the AUCs of Urea, Crea, and UA were 0.552 (*P* > 0.05), 0.663 (*P* < 0.05) and 0.581 (*P* > 0.05), respectively; the sensitivities were 17.0%, 98.1%, and 86.8%, respectively, and the specificities were 95.7%, 30.4%, and 34.8%, respectively, with only Crea being statistically significant, as shown in Figure 3B and Table 4. The AUCs of TB-DOT and T-SPOT.TB for the diagnosis of UTB were 0.792 (*P* < 0.001) and 0.797 (*P* < 0.001), respectively, with a sensitivity of 81.3%, and 90.6%, respectively, and a specificity of 71.4% and 68.8%, respectively, both of which were statistically significant for the diagnosis of UTB, as shown in Figure 3C and Table 3. In addition to this, the AUC of WBC (*P* < 0.01), T-SPOT.TB (*P* < 0.05) and TB-DOT (*P* < 0.05) were significantly increased compared to Urea, see Figure 3D.

**Table 4:** ROC curve for the diagnosis of UTB with a single test.

**Examination/index**	**Cutoff**	**AUC**	***P***	**Sensitivity**	**Specificity**	**95% CI**
WBC	>31.50	0.847^***^	< 0.001	0.911	0.696	0.756–0.939
RBC	>17.00	0.744^***^	< 0.001	0.750	0.783	0.622–0.867
PH	>6.250	0.667^*^	0.020	0.500	0.739	0.530–0.805
Urea	>7.500	0.552^N.S.^	0.473	0.170	0.957	0.417–0.688
Crea	>51.50	0.663^*^	0.024	0.981	0.304	0.524–0.803
UA	>261.5	0.581^N.S.^	0.263	0.868	0.348	0.433–0.730
T-SPOT.TB	>98.00	0.792^***^	< 0.001	0.813	0.714	0.668–0.918
TB-DOT	>0.500	0.797^***^	< 0.001	0.906	0.688	0.647–0.947

Compared to Non-UTB, ^N.S.^*P* > 0.05, ^*^P < 0.05, ^***^P < 0.001; T-SPOT.TB, T-cell spot tests for tuberculosis infection; TB-DOT, tuberculosis antibody test; Crea, creatinine; UA, uric acid; AUC, Area Under Curve; PH, the potential of hydrogen; WBC, white blood cell; RBC, red blood cell; CI, confidence interval.

### Correlation analysis of T-SPOT.TB and TB-DOT with urinary routine and crea

**Figure 4:**
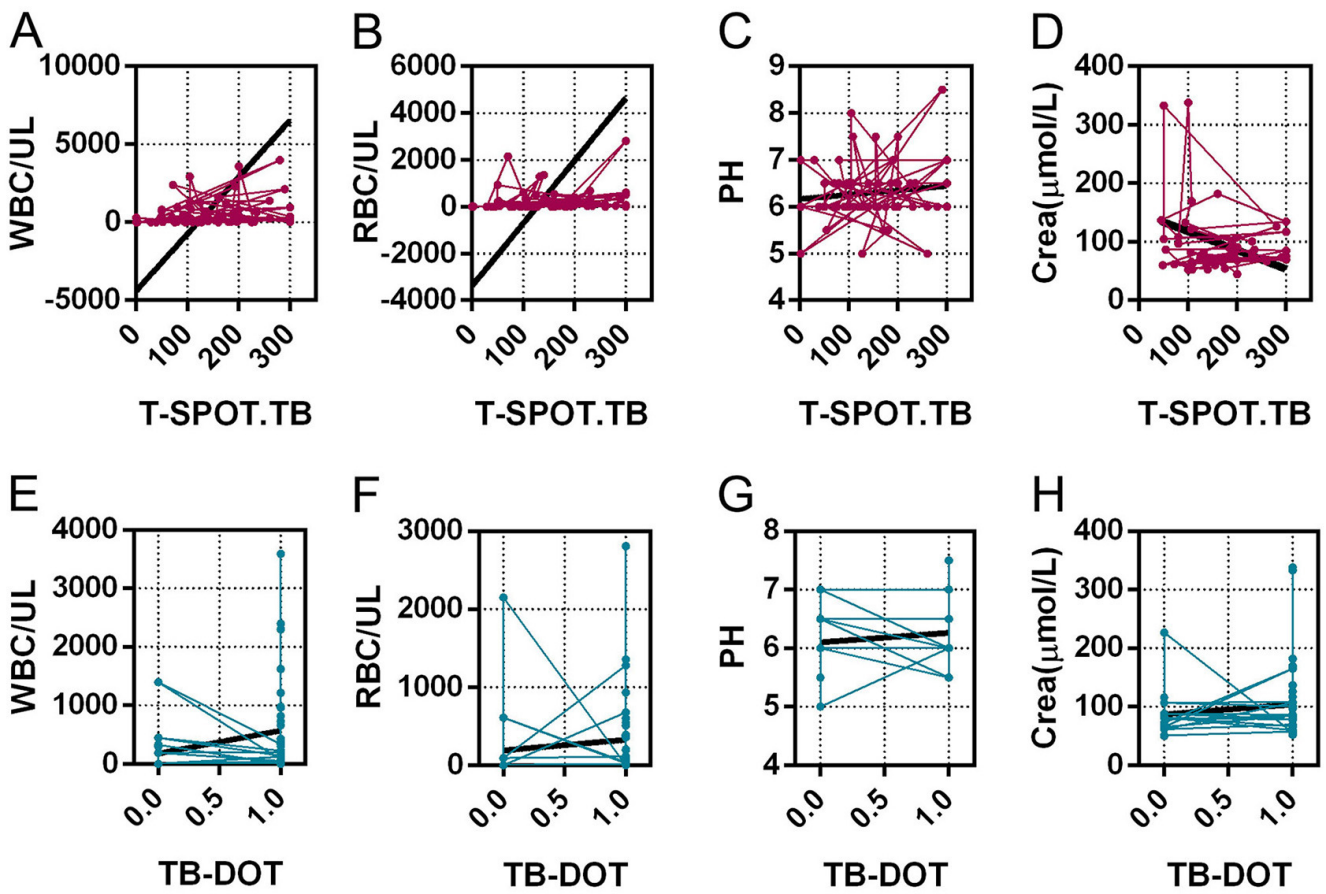
Correlation analysis of T-SPOT.TB and TB-DOT with urinary routine and Crea.

By analyzing the correlation of urinary routine and Crea with TB-DOT and T-SPOT.TB, we concluded that T-SPOT.TB was positively correlated with WBC(*r* = 0.270, *P* < 0.05), negatively correlated with Crea (*r* = −0.201, *P* < 0.05), positively correlated with RBC but not statistically significant (*r* = 0.204, *P* > 0.05), and not correlated with PH (*r* = 0.128, *P* > 0.05) (see Figures 4A–D and Table 5). TB-DOT showed a positive but not statistically significant correlation with WBC (*r* = 0.251, *P* > 0.05) and no correlation with RBC, Crea, and PH (*r* = 0.113, 0.142, 0.128, *P* > 0.05) (see Figures 4E–H and Table 5).

**Table 5:** Correlation analysis of T-SPOT.TB and TB-DOT with urinary routine and Crea.

**Examination**		**WBC**	**RBC**	**PH**	**Crea**
T-SPOT.TB	Pearson r	0.270	0.204	0.128	−0.201
	*P*	0.025	0.093	0.294	0.040
TB-DOT	Pearson r	0.251	0.113	0.142	0.128
	*P*	0.082	0.440	0.312	0.385

T-SPOT.TB, T-cell spot tests for tuberculosis infection; TB-DOT, tuberculosis antibody test; Crea, creatinine; PH, the potential of hydrogen; WBC, white blood cell; RBC, red blood cell; CI, confidence interval.

### Comparison of the diagnostic efficacy of the combined test for UTB

In the WBC parallel combined TB-DOT and T-SPOT.TB assays, the specificity and PPV of WBC+T-SPOT.TB (100.0%, 100.0%) were higher than Crea+T-SPOT.TB (40.8%, 35.6%) with statistical significance (*P* < 0.05); the sensitivity of WBC+TB-DOT+T-SPOT.TB (93.1%) was higher than that of WBC+TB-DOT (90.3%), and its specificity and PPV (77.8%, 87.1%) were lower than those of WBC+T-SPOT.TB (100.0%, 100.0%), while its NPV (87.5%) was higher than that of WBC+T-SPOT.TB (81.0%), which was statistically significant (*P* < 0.05); and the specificity and PPV of WBC+TB-DOT+T-SPOT.TB (77.8%, 87.1%) was significantly higher than those of Crea+TB-DOT+T-SPOT.TB (45.7%, 36.7%), with statistically significant differences (*P* < 0.05) (see Table 6).

**Table 6:** Comparison of the diagnostic efficacy of the combined test for UTB [%, *n*].

**Examination/index**		**Sensitivity**	**Specificity**	**PPV**	**NPV**	χ^**2**^ **test**		**OR [95% CI]**
						χ^2^	***P***	
WBC+	T-SPOT.TB	91.3 (42/46)	100.0 (17/17)^③^	100.0 (42/42)^③^	81.0 (17/21)	46.565	0.000	11.500 [4.508–29.334]
	TB-DOT	90.3 (28/31)	81.3 (13/16)	90.3 (28/31)	81.3 (13/16)	24.076	0.000	40.444 [7.169–228.184]
	T-SPOT.TB+TB-DOT	93.1 (27/29)^②⑥^	77.8 (14/18)^①⑤⑥^	87.1 (27/31)^①⑤⑥^	87.5 (14/16)^①⑥^	24.851	0.000	47.250 [7.687–290.449]
Crea+	T-SPOT.TB	94.1 (16/17)	40.8 (20/49)	35.6 (16/45)	95.2 (20/21)	7.100	0.008	11.034 [1.353–90.026]
	TB-DOT	91.0 (10/11)	88.2 (15/17)^③^	83.3 (10/12)^③^	93.8 (15/16)	17.082	0.000	75.000 [5.973–941.798]
	T-SPOT.TB+TB-DOT	100.0 (11/11)^②④^	45.7 (16/35)	36.7 (11/30)	100.0 (16/16)^①②④^	7.710	0.005	0.543 [0.401–0.736]
WBC+Crea+TB-DOT+T-SPOT.TB		35.1 (13/37)^⑤^	100.0 (16/16)^③^	100.0 (13/13)^③^	40.0 (16/40)^⑤^	7.449	0.006	1.542 [1.216–1.954]

① Compare to WBC+T-SPOT.TB, *P* < 0.05; ② Compare to WBC+TB-DOT, *P* < 0.05; ③ Compare to Crea+T-SPOT.TB, *P* < 0.05; ④ Compare to Crea+TB-DOT, *P* < 0.05; ⑤ Compare to Crea+T-SPOT.TB+TB-DOT, *P* < 0.05; ⑥ Compare to WBC+Crea+TB-DOT+T-SPOT.TB, *P* < 0.05; T-SPOT.TB, T-cell spot tests for tuberculosis infection; TB-DOT, tuberculosis antibody test; Crea, creatinine; UA, uric acid; PPV, Positive predictive value; NPV, Negative predictive value; WBC, white blood cell; CI, confidence interval; OR, odds ratio.

In the Crea combined TB-DOT and T-SPOT.TB assays, the specificity and PPV of Crea+TB-DOT (88.2%, 83.3%) were higher than those of Crea+T-SPOT.TB (40.8%, 35.6%), the sensitivity of Crea+TB-DOT+T-SPOT.TB (100%) was higher than that of Crea+TB-DOT (91.0%) and WBC+TB-DOT (90.3%), and the NPV of Crea+TB-DOT+T-SPOT.TB (100%) was higher than Crea+TB-DOT (93.8%), WBC+T-SPOT.TB (81.0%) and WBC+TB-DOT (81.3%), all with statistically significant (*P* < 0.05) (see Table 6).

The sensitivity and NPV of the parallel combination of WBC+Crea+TB-DOT+T-SPOT.TB were only 35.1% and 40.0%, which were lower than any of the parallel combination assays and statistically different from Crea+TB-DOT+T-SPOT.TB (*P* < 0.05); its specificity and PPV, although both were 100.0%, were only different from Crea+T-SPOT.TB with a statistically significant difference (*P* < 0.05) (see Table 6).

### ROC curve of the combined test for the diagnosis of UTB

**Figure 5:**
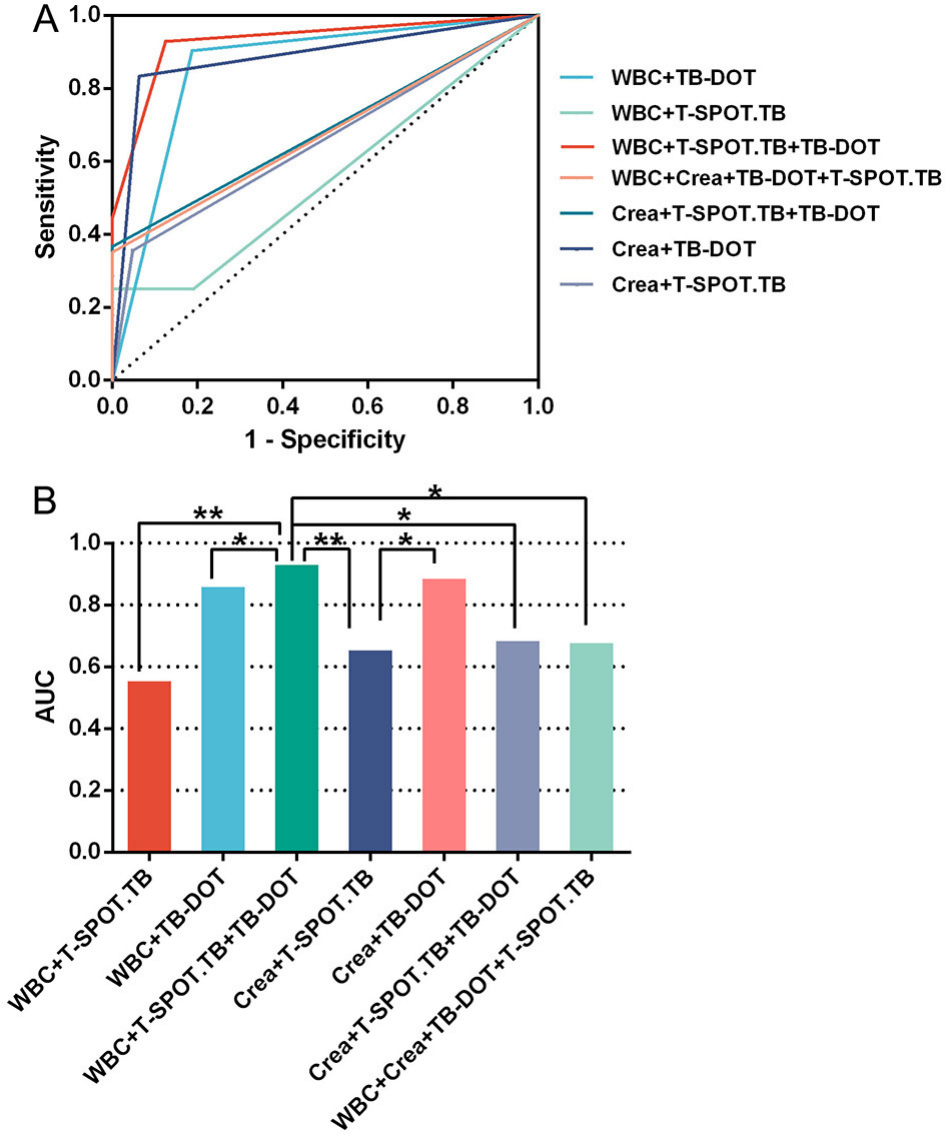
ROC curve of the combined test for the diagnosis of UTB.

In multiple parallel combined assays, WBC+T-SPOT.TB, WBC+TB-DOT, WBC+TB-DOT+T-SPOT.TB, Crea+T-SPOT.TB, Crea+TB-DOT, Crea+TB-DOT+T-SPOT.TB, WBC+Crea+TB-DOT+T-SPOT.TB with AUCs of 0.554, 0.858, 0.930, 0.654, 0.885, 0.683, 0.676, respectively, where WBC+TB-DOT+T-SPOT.TB had the largest AUC value and was significantly higher than WBC+T-SPOT.TB (*P* < 0.01), WBC+TB-DOT (*P* < 0.05), Crea+T-SPOT.TB (*P* < 0.01), Crea+TB-DOT+T-SPOT.TB (*P* < 0.05), and WBC+Crea+TB-DOT+T-SPOT.TB (*P* < 0.05) addition to this, The sensitivity of WBC+TB-DOT+T-SPOT.TB was 92.9%, which was also the highest (see Table 7 and Figure 5).

**Table 7:** ROC curve of the combined test for the diagnosis of UTB.

**Examination/index**		**Cutoff**	**AUC**	***P***	**Sensitivity**	**Specificity**	**OR [95% CI]**
WBC+	T-SPOT.TB	>13.50	0.554	0.471	0.250	1.000	0.417–0.690
	TB-DOT	>0.500	0.858	< 0.001	0.903	0.813	0.730–0.986
	T-SPOT.TB+TB-DOT	>0.500	0.930	< 0.001	0.929	0.875	0.863–0.997
Crea+	T-SPOT.TB	>0.500	0.654	0.045	0.356	0.952	0.521–0.787
	TB-DOT	>0.500	0.885	< 0.001	0.833	0.938	0.741–1.029
	T-SPOT.TB+TB-DOT	>0.500	0.683	0.043	0.367	1.000	0.532–0.834
WBC+Crea+TB-DOT+T-SPOT.TB		>0.500	0.676	0.044	0.351	1.000	0.533–0.819

T-SPOT.TB, T-cell spot tests for tuberculosis infection; TB-DOT, tuberculosis antibody test; Crea, creatinine; UA, uric acid; AUC, Area Under Curve; WBC, white blood cell; CI, confidence interval; OR, odds ratio.

## Discussion

China is among the 30 countries with the greatest incidence of TB worldwide, with around 800,000 new cases recorded annually. Among legally recognized infectious diseases, TB is the second most common, making prevention and control of the disease a critical issue (World Health Organization, 2022a,b). To meet the goals of our Action Plan to Stop TB (2019–2022), which calls for lowering the national incidence of TB to less than 55 cases per 100,000 individuals and lowering the mortality rate to less than 3 deaths per 100,000 by 2022, we need to improve screening to find patients as soon as possible. Furthermore, to avoid problems like bladder contracture, hydronephrosis, and spontaneous bladder rupture brought on by UTB, physicians require a quick.

A typical urine test, such as glucose, ketone bodies, protein, occult blood, RBC, WBC, and amino acids assess numerous substances. It's crucial to remember that elevated WBC counts do not always signify kidney tuberculosis (TB), even if they are frequently linked to the condition. Alternatively, they may also be a reaction to infections brought on by harmful bacteria in the urinary system (Wang et al., 2021). The current study found that MTB infection led to an increase in WBC, which is consistent with the findings of the study by Shah et al. (2022). The microscopic WBC count was significantly higher (717.7 ± 933.5) and the WBC positivity rate for UTB was 94.6%, significantly higher than that of non-UTB at 52.2%, *P* < 0.001. Hematuria is another important symptom of UTB that happens between 70% and 80% of the time. It is often accompanied by painful urination, urgency, and frequency of urinating. Although kidney disease can also induce hematuria, bladder lesions are usually the source. In this study, the RBC positivity rate of UTB was 71.4%, significantly greater than the non-UTB positivity rate of 21.7%, *P* < 0.001, and the number of microscopic WBCs was much higher (252.6 ± 460.9). While UTB patients may exhibit some abnormalities in their urine routine, these abnormalities are non-specific and can also occur in other kidney-related conditions. Therefore, UTB cannot be ruled out by abnormalities in urine routine, but it may manifest in conjunction with them. Additionally, we discovered in this study that WBC had a specificity of 78.6% but an NPV of just 47.8%, which was not a good diagnostic value for UTB.

UTB is almost always secondary to pulmonary TB infection. UTB will manifest after MTB enters the bloodstream and enters the kidneys. If the patient's resistance is still weak at this time, the disease will develop rapidly, and in serious cases, it will lead to bilateral kidney lesions, which will cause Crea, UA, and Urea to increase (Kim et al., 2012). In the present study, Urea (5.66 ± 2.67), Crea (96.03 ± 56.22) and UA (395.04 ± 143.70) levels were elevated in UTB compared to non-UTB, and the positive rate of Crea was significantly higher, *P* < 0.05. Urea levels may also increase in other situations, such as upper gastrointestinal bleeding (Krüger et al., 2008). Although abnormalities in Crea, UA, and Urea occur in patients with UTB, the diagnosis of UTB requires a comprehensive judgment. If the Crea is high, it is possible that UTB does not cause it. If the kidney function was normal before, and the Crea is high after having UTB, the kidney function is likely destroyed by UTB, which causes the Crea to rise. As a result, it is not suggested to diagnose UTB purely based on impaired renal function.

TB-DOT, a serological test, TB antibody measurement, positive for having been infected or currently infected with MTB (Nsubuga et al., 2023). In this study, the positivity rate of TB-DOT was 87.88%, which was significantly higher than that of non-UTB, *P* < 0.001; its sensitivity and specificity were 85.7% and 78.6%, respectively, and its diagnostic efficacy was still not high.

T-SPOT.TB is also commonly used to diagnose UTB; MTB-specific T cells specifically secrete γ-interferon. The T-SPOT.TB test is based on the principle of γ-interferon release assay (TB-IGRA), an enzyme-linked immunospot technique for the specific detection of TB effector T cells in the subject to diagnose whether the subject is infected with MTB. The test has numerous uses and is early, quick, affordable, and biosecurity. Moreover, it has a very high sensitivity and detection rate in diagnosing TB, according to existing research, with a sensitivity and specificity of 84.0% and 99.1% in children with TB and 83.7% and 80.2% in patients with co-infected HIV TB, respectively (Stout et al., 2018; Lee et al., 2013). The sensitivity and specificity of the T-SPOT.TB in this study were 77.4% and 100.0%, respectively, which is more consistent with the findings of Liao et al. (2009), but its diagnostic efficacy was still not particularly high. However, studies have shown that peripheral blood T-SPOT.TB has its own defects and is susceptible to the influence of peripheral blood T-lymphocyte count (Bocchino et al., 2008), which often leads to false-negative results. The sensitivity of T-SPOT.TB is constrained by the fact that several non-TB pathogen infections also encourage T cells to secret interferon, and individuals with the latent and infected phases of the disease are difficult to diagnose. Although each of these tests has pros and cons of its own, they are all helpful in the early identification of UTB.

The results of this study showed that, among the tests, the positive rate and specificity of Urea and UA were too low; the sensitivity of Crea, RBC and PH was average, but the specificity was poor; the sensitivity and specificity of WBC and TB-DOT were average; and the sensitivity of T-SPOT.TB was poor, but it had a high positive rate and specificity, which showed that each test was diagnostic of UTB efficacy was not high (Figure 2, Tables 2, 3). And, by correlation analysis, urinary routine as long as WBC was correlated with T-SPOT.TB (*r* = 0.270, *P* < 0.05) and TB-DOT (*r* = 0.251, *P* > 0.05) and was not statistically significant with TB-DOT; Crea in renal function was only correlated with T-SPOT.TB (*r* = −0.201, *P* < 0.05) (see Figure 4 and Table 5). However, in the parallel combined assay, WBC+TB-DOT+T-SPOT.TB had a slightly lower sensitivity (93.1%) than Crea+T-SPOT.TB (94.1%), but its specificity and PPV (77.8%, 87.1%) were higher than Crea+T-SPOT.TB (40.8%, 35.6%); the sensitivity of Crea+TB-DOT+T-SPOT.TB (100.0%) was the highest, but its specificity (45.7%) was lower; and the specificity of WBC+Crea+TB-DOT+T-SPOT.TB (100.0%) was higher but its sensitivity (35.1%) was lower. Additionally, as shown in Figures 2, 4 and Tables 4, 7, the AUC of WBC+TB-DOT+T-SPOT.TB (0.930) was noticeably greater than that of any of the individual tests or other analogous combination tests. Therefore, this study suggests that the practical significance of combining the three methods of WBC in urine and TB-DOT and T-SPOT.TB in blood for diagnosis is greater. However, the focus of this trial analyzed the laboratory diagnosis of UTB and did not analyze drug resistance in UTB patients, which will be the focus of our next study.

Therefore, for the diagnosis and treatment of UTB, laboratory testing is crucial. Numerous new detection techniques have arisen in recent years because of the advancement of molecular biology. A single detection method has a low detection effect, but the combined use of several detection methods can increase the accuracy of its detection, reduce the time it takes to detect, and make it easier to diagnose and treat UTB early.

In conclusion, in the early identification of UTB, the sensitivity of T-SPOT.TB or TB-DOT tests are much higher than urine routine and renal function tests. The parallel combination of WBC, TB-DOT, and T-SPOT.TB has better diagnostic efficacy for UTB, which is beneficial for rapid clinical diagnosis of UTB.

## Data Availability

The datasets presented in this study can be found in online repositories. The names of the repository/repositories and accession number(s) can be found in the article/supplementary material.
